# Formation of Secondary and Tertiary Volatile Compounds Resulting from the Lipid Oxidation of Rapeseed Oil

**DOI:** 10.3390/foods10102417

**Published:** 2021-10-12

**Authors:** Sandra Grebenteuch, Lothar W. Kroh, Stephan Drusch, Sascha Rohn

**Affiliations:** 1 Department of Food Chemistry and Analysis, Institute of Food Technology and Food Chemistry, Technische Universität Berlin, Gustav-Meyer-Allee 25, 13355 Berlin, Germany; sandra.grebenteuch@tu-berlin.de (S.G.); lothar.kroh@tu-berlin.de (L.W.K.); 2 Institute for Food and Environmental Research e. V., Papendorfer Weg 3, 14806 Bad Belzig, Germany; 3 NutriAct-Competence Cluster Nutrition Research, c/o The German Institute of Human Nutrition Potsdam-Rehbrücke, Arthur-Scheunert-Allee 114-116, 14558 Nuthetal, Germany; stephan.drusch@tu-berlin.de; 4 Department of Food Technology and Food Material Science, Institute of Food Technology and Food Chemistry, Technische Universität Berlin, Königin-Luise-Str. 22, 14195 Berlin, Germany

**Keywords:** tertiary products, lipid-derived aldehydes, volatile compounds, flavour deterioration, hexanal, 2-butyl-2-octenal, aldol condensation, alkyl furans, 2-pentylfuran, thermal oxidation

## Abstract

The lipid oxidation of fats and oils leads to volatile organic compounds, having a decisive influence on the sensory quality of foods. To understand formation and degradation pathways and to evaluate the suitability of lipid-derived aldehydes as marker substances for the oxidative status of foods, the formation of secondary and tertiary lipid oxidation compounds was investigated with gas chromatography in rapeseed oils. After 120 min, up to 65 compounds were detected. In addition to secondary degradation products, tertiary products such as alkyl furans, ketones, and aldol condensation products were also found. The comparison of rapeseed oils, differing in their initial peroxide values, showed that the formation rate of secondary compounds was higher in pre-damaged oils. Simultaneously, a faster degradation, especially of unsaturated aldehydes, was observed. Consequently, the formation of tertiary products (e.g., alkyl furans, aldol adducts) from well-known lipid oxidation products (i.e., propanal, hexanal, 2-hexenal, and 2-nonenal) was investigated in model systems. The experiments showed that these compounds form the new substances in subsequent reactions, especially, when other compounds such as phospholipids are present. Hexanal and propanal are suitable as marker compounds in the early phase of lipid oxidation, but at an advanced stage they are subject to aldol condensation. Consequently, the detection of tertiary degradation products needs to be considered in advanced lipid oxidation.

## 1. Introduction

Lipid oxidation is one of the most dominant chemical processes which takes place during the lifetime of foods. Besides the availability of oxygen and UV light, temperature and heating time are the most important external influence parameters. Among the endogenous factors that influence the course of lipid oxidation are the concentration of free fatty acids and antioxidants, the degree of saturation, as well as the food matrix, water content, and further prooxidants [1,2]. As a consequence, essential polyunsaturated fatty acids [3,4] are particularly prone to oxidation and their presence in foods is associated with reduced shelf-life and changes in their sensory perception [5,6,7,8]. For this reason, it is important to monitor lipid oxidation in fats and oils, as well as corresponding foods.

During lipid oxidation hydroperoxides are formed as primary reaction products. However, they are odourless and tasteless, but react to a variety of secondary degradation products of different compound classes [9]. Especially, volatile organic compounds (VOC) such as aldehydes, alkanes, alcohols, esters, and epoxides are formed due to the β-cleavage of the hydroperoxides and are generally seen as secondary degradation products of lipid oxidation [10]. However, as these secondary compounds are still reactive due to their chemical structure, they can react independently or with other compounds. Accordingly, tertiary VOCs such as the methyl ketones of 2,4-alkadienals can also form during lipid oxidation [11]. Most of these VOC are aroma-active and can cause significant sensory changes [12,13]. In addition to unpleasant sensory qualities, some VOC can also have a negative impact on human health. This is especially valid for the unsaturated 2-alkenals and 2,4-alkadienals [14,15,16].

Lipid oxidation has been well studied and several markers are already used for an analytical evaluation, especially at temperatures below 100 °C. A very simple but most prominent estimate is the peroxide value (POV). It is generally used in routine analysis to determine the oxidative status of oils. However, this estimate and other frequently used methods such as the anisidine value, the photometric determination of the malondialdehyde content or the measurement of conjugated dienes, have disadvantages and their use is often limited to specific applications or simple routine analyses [17]. With regard to secondary lipid oxidation products, hexanal is the most prominent marker for the progress of lipid oxidation, especially when evaluating ω-6 fatty acids [18,19], while, propanal is a typical degradation product of ω-3 fatty acids [20]. Additionally, combinations and ratios of different VOC are also described as indicators [21]. Besides oxidative lipid degradation reactions, polymerization reactions and hydrolytic cleavage are of significance and can lead to polar compounds such as mono-and diacylglycerols, free fatty acids, and polymerized triacylglycerols.

So far, studies frequently focused on the traditional and individual markers such as hexanal, propanal [20,22]. Further, primary products such as (hydro)peroxides [23,24] or other non-volatile final products such as polymers or malondialdehyde were evaluated [25]. In addition, many authors analysed and listed volatile compounds that were formed during lipid oxidation without considering the course of formation and degradation of the single VOC or the course of the individual compound classes in general [9,14,22]. Furthermore, there is a lack of knowledge about tertiary degradation products of the lipid oxidation. Here, it seems to be important to take possible interactions with other food compounds into account. Nucleophilic nitrogen containing compounds (e.g., phospholipids) can affect composition of the VOC, as well [11,26,27]. For these reasons, the monitoring of tertiary compounds from lipid oxidation can be a superior and simple method to the traditional methods used in routine analysis as described above.

In addition, food processing has undergone significant regulations in recent years. In order to reduce the formation of process-induced contaminants, sometimes also called foodborne toxicants, such as *trans*-fatty acids [23], acrylamide [28], chloropropanols, and furanes [29] heating temperatures and times were reduced. For example, frying oils should be kept below 175 °C, as the formation of acrylamide is strongly accelerated at temperatures higher than 175 °C [30]. However, under domestic conditions, oils are continuously “aging”, because of being often stored under poor conditions for several weeks or even months and thus, being susceptible to lipid oxidation. Further, the regulations and recommendations mentioned above cannot be easily controlled by an average chef and/or consumer. Consequently, excessive heating (such as in inappropriate frying/deep-frying), reheating of foods, or bad storing, cannot be excluded. Marker compounds can be applied to identify inappropriate use of temperature, excessive heating times, and are helpful in improving the characterisation of the oxidative status of an oil. As many chemical methods for the characterisation of non-volatile compounds as markers are time-consuming and require a lot of chemicals, the analysis of volatile compounds is particularly promising [31]. Recent studies also show developments to assess lipid oxidation by volatile compounds online during frying [32].

The aim of the present study was to investigate the oxidative stability of ω-3 and ω-6 fatty acids at elevated temperatures. Commercially available cold-pressed rapeseed oils were investigated as their fatty acid composition (ω-6/ω-3) is highly appreciated and health benefits are associated [33,34,35]. For comparison, a fresh rapeseed oil and an artificially aged oil were used. In addition to the characterisation of secondary VOC, the formation of tertiary degradation products was also characterised. In order to investigate the subsequent degradation reactions of VOC in more detail, individual compounds were analysed in model experiments with and without the addition of food relevant compounds, in particular phospholipids such as lecithin, as their intense involvement in oxidation reactions has been already proven [36].

## 2. Materials and Methods

### 2.1. Samples

Two commercially available cold-pressed rapeseed oils from a local supermarket were used: a fresh rapeseed oil with an initial POV of 4 mEq O_2_/kg (‘RO 4’) and an aged rapeseed oil with a POV of 20 mEq O_2_/kg (‘RO 20’). To achieve the second oil, ‘RO 4’ was opened and stored at room temperatures for 6 months until a peroxide value (POV) of 20 could be measured. 15 mEq O_2_/kg is the recommended maximum POV value for cold-pressed oils [37].

### 2.2. Chemicals and Materials

Propanal, pentanal, hexanal, heptanal, octanal, nonanal, *trans*-2-butenal, *trans*-2-pentenal, *trans*-2-hexenal, *trans*-2-heptenal, *trans*-2-octenal, *trans*-2-nonenal, *trans*-2-decenal, *trans*-2-undecenal (≥95%), *trans*,*trans*-2,4-heptadienal, *trans*,*trans*-2,4-decadienal, 1-penten-3-ol, 2-heptanone, 2-octanone, 2-pentylfuran, 2-ethylfuran, 2-butyl-2-octenal, 2-methyl-2-pentenal, propanoic acid, caproic acid, iron(II) chloride (tetrahydrate), iron(III) nitrate (nonahydrate), and iron(II) sulphate (heptahydrate) were obtained from Merck KGaA, Darmstadt, Germany. Acetic acid, chloroform, potassium iodide and acetonitrile were purchased from Carl Roth GmbH + Co. KG, Karlsruhe, Germany. Sodium thiosulphate (0.1 N) was purchased from Bernd Kraft GmbH, Duisburg, Germany. 1,2-Dipalmitoyl-*sn*-glycero-3-phosphoethanolamine (PE, >96%), and 1,2-dipalmitoyl-*sn*-glycero-3-phosphocholine (PC) were obtained from TCI Chemicals Europe N.V., Zwijndrecht, Belgium. Paraffin oil (Pfeiffer^®^ P3) was purchased from MasCom Technologies GmbH, Bremen, Germany. All of the chemicals were of analytical grade, if not mentioned otherwise.

### 2.3. Preparation of Model Systems and Experiments with Oils 

VOC from rapeseed oils were analysed by headspace gas chromatography-mass spectrometry (HS-GC-MS) as described previously [11]. Briefly, 1 g of oil sample was weighed into a 20 mL-headspace vial and incubated at 160 °C for 5–120 min. In model experiments, the single aldehydes propanal, hexanal, 2-hexenal, and 2-nonenal were dissolved in inert paraffin oil as an oily matrix with a final concentration of 0.01% for 2-alkenals and 1% for aldehydes. One gram of this solution was weighed into a 20 mL-headspace vial and mixed for 15 s. Optionally, 10 mg of iron (II) chloride or PE, PC, H_2_O, propanoic acid, or caproic acid, were added directly to the solution in the headspace vial. The model systems with propanal and hexanal were extracted with 1 mL acetonitrile eith mixing for 15 s and incubating for 30 min. Finally, the upper acetonitrile phase was transferred into a simple GC vial. The samples of the model experiments were heated at 160 °C for 0–60 min. The experiments with the 2-alkenals were also analysed by HS-GC-MS. Due to the lower volatility of the oligomeric aldol products, the experiments with the other single aldehydes were analysed with liquid injection gas chromatography mass spectrometry (GC-MS).

### 2.4. Determination of the Peroxide Value (POV)

As a traditional and indicative method, POV was determined according to Wheeler [38] in a microanalysis approach. One-hundred milligrams of rapeseed oil was weighed into an Erlenmeyer flask. A total of 30 mL of solvent (acetic acid/chloroform 3:2) and 0.5 mL of saturated potassium iodide solution were added. After stirring for 60 s, 30 mL of distilled water were added and titrated potentiometrically (TitroLine^®^ 7000, Pt 61 electrode, Xylem Analytics Germany Sales GmbH & Co. KG, Weilheim, Germany with sodium thiosulphate (0.001 N). The titration endpoint was detected by automated titration with regard to the strong change in electrochemical potential.

### 2.5. VOC Analysis with Static Headspace GC-MS (HS-GC-MS)

For an automated sample incubation and application, the GC-MS system was equipped with a Combi-PAL-RSI autosampler from Axel Semrau GmbH & Co. KG, Sprockhövel, Germany. The agitator module incubated (160 °C) and agitated (250 rpm) the samples prior to GC injection. Subsequently, 1 mL of vapor space was injected into the GC−MS system, consisting of a GC-17A gas chromatograph and a QP5000 mass detector (both Shimadzu Deutschland GmbH, Duisburg, Germany). VOC were separated using a Rtx^®^-Volatiles column (60 m × 0.25 mm, 1 μm, Restek GmbH, Bad Homburg, Germany). The following settings were used: carrier gas, helium; flow 1.00 mL/min; split 1:20; injection temperature 230 °C; interface temperature 230 °C; ion source temperature 200 °C; ionization energy 70 eV; temperature gradient 40 °C for 5 min, 10 °C/min to 150 °C, 2 °C/min to 210 °C. Under these conditions, it is possible to separate structural isomers such as hexanal and 2-hexanone.

### 2.6. GC-MS Analysis

The acetonitrile extracts of the aldehyde experiments were analysed with an automated injection using an autosampler (AOC-20i, Shimadzu Deutschland GmbH, Duisburg, Germany). One microliter was injected into the GC-MS system consisting of a GC-2010 and a GCMS-QP2010 plus mass detector (both Shimadzu Deutschland GmbH, Duisburg, Germany). The analytes were separated using a DB-23 60 m × 0.25 mm × 0.25 μm (Agilent Technologies Inc., Santa Clara, CA, USA). The following settings were used: carrier gas, helium; flow 1.50 mL/min; split 1:20; injection temperature 250 °C; interface temperature 250 °C; ion source temperature 200 °C; ionization energy 70 eV; solvent cut time 3.5 min; temperature gradient 60 °C, 10 °C/min to 240 °C, for 5 min. 

### 2.7. Chemical Identification with GC-MS

Chemical identification was conducted by comparing retention times and mass spectra of samples with those of analytical standards and using the NIST (National Institute of Standards and Technology) database, except for the aldol trimer 2,4-dimethyl-2,4-heptadienal, which was only confirmed by NIST database. The quantitation was performed in SCAN mode (headspace: mass scan *m/z* 33–350; liquid injection: mass scan *m/z* 33–750) using total ion current (TIC) and expressed as abundance units (AU) and provide semiquantitative data. Data acquisition was performed using the GCMSsolution software version 1.20 and version 2.71 (Shimadzu Deutschland GmbH, Duisburg, Germany).

### 2.8. Statistical Analysis

The samples were prepared in triplicate for each heating time. The values of POV were analysed four times for each sample. All of the results are shown as means ± standard deviation. The data were analyzed using a two-way analysis of variance (ANOVA) to determine the effect of reactants on the degradation aldehydes followed by a Dunnett post-hoc test. Graphical evaluations were carried out using GraphPad Prism 8.0.2 software (San Diego, CA, USA).

## 3. Results and Discussion

### 3.1. POV and Hexanal

**Figure 1:**
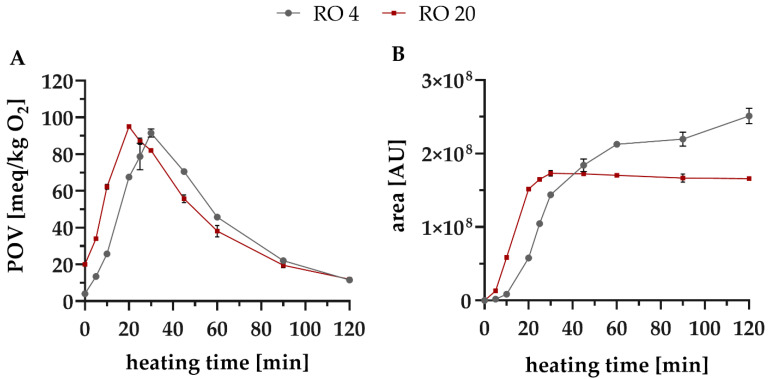
(**A**): POV and (**B**): hexanal content of RO4 (●) and RO20 (▪) heated at 160 °C for 120 min.

The measurement of the hydroperoxides as POV in mEq/kg O_2_ is a common estimate to determine the oxidative status of an oil, especially in routine analysis. Various authors used POV to study the oxidation status of deep-frying oils [39,40,41]. When comparing the data, it must be noted that the POV can be determined with different methods, including iodometric assays or iron complexing-based methods [42]. The automated potentiometric titration used in the present study ensures the precise and reproducible determination of the POV, even with small sample volumes, when the waiting and titration times are strictly followed [43,44]. The determination of the POV of intensively heated oils is not appropriate because of the instability of the hydroperoxides, especially at elevated temperatures (>130 °C) [40,45,46]. However, peroxides are continuously formed, even when the oil is cooling down [40,47]. The concentration of peroxides also depends on the pre-damage of an oil. In the present study, depending on the initial POV, it further increased with a time lag up to a maximum value of 92 mEq/kg O_2_ after 30 min for ‘RO 4’ and 95 mEq/kg O_2_ after 20 min for ‘RO 20’ (Figure 1). When the maximum was reached, POV steadily decreased and after 90 min of heating time, there was no difference between POV of both rapeseed oils (Figure 1A). In the ‘RO 20’ oil, there was no significant difference of POV after 10 and 45 min, due to the continuous formation and decomposition of the hydroperoxides (Figure 1A). As already pointed out in the literature, POV is not suitable as a marker for the oxidative status of oil samples, because of severe limitations: during storage, POV of highly oxidized samples is decreasing from a certain time point, as the peroxides decompose faster than they are formed resulting in low a POV. Consequently, more reliable markers covering the whole course of lipid oxidation are necessary to evaluate the oxidative status of oil samples. As described later in detail, tertiary products such as alkyl furans or aldol reaction products should be considered instead.

Formation of the secondary degradation products from the lipid oxidation occurs after a certain time lag to peroxide formation. As soon as more peroxides were decomposed than formed (after reaching the max. POV), the rate of formation of hexanal decreased (Figure 1B). This indicates subsequent degradation reactions of hexanal. This is due to aldol reactions leading to tertiary degradation products, which derive from the secondary products of the lipid oxidation by decomposition (compare to Section 3.3.2) and/or further reaction with other compounds present. It is important to clearly separate secondary from tertiary degradation products, as the presence of tertiary products is an indication for a strongly advanced lipid oxidation.

### 3.2. Formation of Volatile Organic Compounds (VOC)

In the present study, formation of VOC has been determined in order to investigate the different courses of lipid oxidation of the two oil samples. After a heating time of 120 min, up to 65 compounds were detected. In addition to secondary degradation products, tertiary degradation products such as alkyl furans, organic acids, and ketones were found [11]. A detailed overview of all volatile compounds is listed in Tables S1–S3. Figure S1A–D additionally shows the HS-GC-MS chromatograms of both oils after 5 min and 120 min heating time. In comparison to the previous studies described in the literature, there was also 50 to 80 different VOC in thermally-stressed rapeseed oil are described [48,49]. From the 65 compounds detected in the present study, 17 compounds with the most intense areas were selected and further divided into the following compound classes: aldehydes, 2-alkenals, 2,4-alkadienals, 2-alkyl furans, and methyl ketones. Expectedly, substances from all major classes of lipid oxidation products were formed, but quantitative differences have been found depending on the age of the oil. Most of all, the well-known marker substance hexanal was formed in the highest quantities (Figure 1B), confirming data from the literature [48,50].

**Figure 2:**
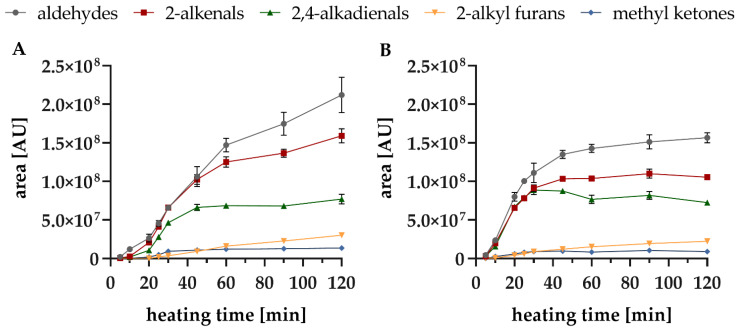
Formation of different compound classes (aldehydes exclude hexanal) during lipid oxidation of rapeseed oils at 160 °C up to 120 min; (**A**): ‘RO 4’; (**B**): ‘RO 20’.

In the aged oil sample ‘RO 20’, all of the compounds were formed earlier and with higher quantities compared to the non-aged oil. For example, after 10 min there was a 7-fold higher amount of hexanal in ‘RO 20’ (Figure 1B). A similar behaviour was observed for further individual compounds, but there were differences between the compound classes in general, mostly depending on heating time. The most significant difference between the two oils was that the rate of formation of aldehydes, 2-alkenals, and 2,4-alkadienals decreased significantly in ‘RO 20’ over time (Figure 2). 

**Figure 3:**
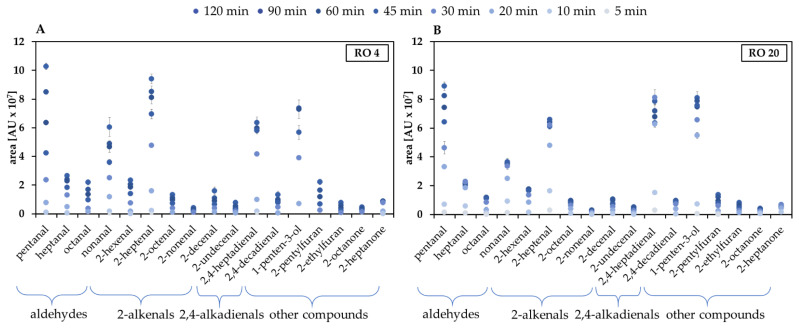
Course of formation of 17 compounds from lipid oxidation over 120 min heated at 160 °C; (**A**): ‘RO 4’ (**B**): ‘RO 20’. The areas of hexanal are presented in Figure 1B.

At the beginning, a faster formation phase of all compounds (except for 2-alkyl furans) up to 60 min could be observed. This strong slope decreased from 60 min, but in ‘RO 4’ the compound classes continued to increase, whereas in ‘RO 20’ a stagnation of the hexanal content (Figure 1B) and all aldehydes in general (Figure 2B) from 30 min was observed. Moreover, content of the 2,4-alkadienales slowly decreased after 30 min. Due to their chemical structure, containing two double bonds, 2,4-alkadienals are still susceptible to further oxidative reactions [50]. Subsequently, contents of aldehydes and 2-alkenals were higher in the fresh oil (‘RO 4’) than in the aged oil (‘RO 20’). As a result of the advanced oxidative stress of ‘RO 20’, those compounds were more unstable and likely to react in follow-up reactions. Tertiary degradation products such as 2-alkyl furans additionally formed, and contents continuously increased. The individual formation rates of the selected 17 VOC over time are shown in Figure 3. In ‘RO 20’ a leap increase between 10 and 20 min was observed (Figure 3B, e.g., 2,4-heptadienal), whereas in ‘RO 4’ a more continuously increase in content was observed only after 30 min (Figure 3A, e.g., 2-heptenal).

However, at the beginning, the attack of the polyunsaturated linolenic acid is not preferred to the double unsaturated linoleic acid, as is the case at lower temperatures [3]. This is obvious from the early formation of degradation products from linoleic acid, such as 2-heptenal and 2,4-decadienal simultaneous with the formation of products from linolenic acid such as 2,4-heptadienal. The individual kinetics are shown in more detail in Figure S2 (provided in Supplementary Materials).

### 3.3. Further Degradation Reactions of Secondary Lipid Oxidation Products to Tertiary Products

As secondary degradation products continuously form during lipid oxidation, a parallel degradation to further compounds is difficult to follow. For this reason, individual compounds from the different compound classes were tested in model systems. In this study, focus was on the compound classes of saturated aldehydes and monounsaturated 2-alkenals, as previous studies have shown that double unsaturated secondary degradation products (2,4-alkadienals) can react to form methyl ketones as tertiary lipid oxidation products [11].

#### 3.3.1. Formation of 2-Alkyl Furans–Degradation of 2-Alkenales in Model Studies

As the two 2-alkyl furans, 2-pentylfuran and 2-ethylfuran, were detected in both rapeseed oil samples, their formation was studied exemplarily by incubating 2-hexenal and 2-nonenal at 160 °C in an inert paraffin oil. However, in these model systems no compounds are present that would initiate radical lipid oxidation, e.g., photosensitizers, enzymes, or reactive oxygen species. Due to this lack of radicals starting and occurring in lipid oxidation, both compounds were comparatively stable (degradation < 10% within 60 min) and no further VOC could be observed. For this reason, iron was used as a radical starter. Three different iron compounds were tested (FeCl_2_, FeSO_4_, Fe(NO_3_)_3_), as in oily matrices, the solubility and dissociation potential of iron salts can be reduced [51]. However, studying the kinetics of the degradation rate of the reactant was most apparent, when iron(II) chloride was used. In contrast, iron(II) sulphate and iron(III) nitrate showed a too intense degradation of the 2-alkenals. Furthermore, nitrate and sulphate are able to undergo redox reactions and to therefore, leading to side reactions.

**Figure 4:**
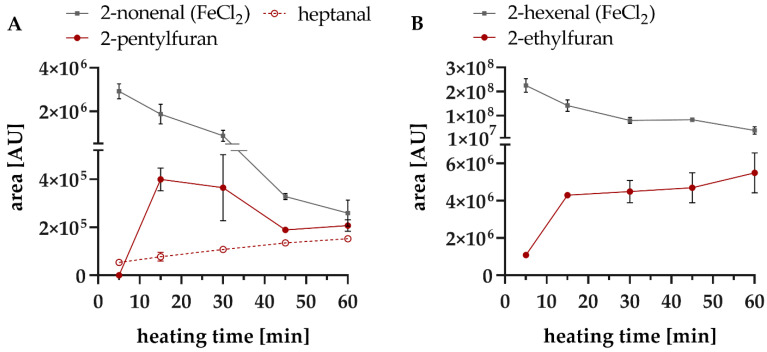
Formation of alkyl furans from the respective 2-alkenales 2-nonenal (**A**) and 2-hexenal (**B**) at 160 °C for 60 min.

The 2-alkenals’ model systems with iron(II) chloride showed a strong degradation of the 2-alkenals to the corresponding 2-alkyl furans (Figure 4).

**Figure 5:**
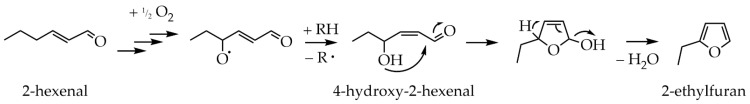
Mechanism of the formation of 2-ethylfuran starting from 2-hexenal (based on Adams et al. 2011).

Adams et al. (2011) proposed a reaction mechanism for the formation of 2-ethylfuran from 2-hexenal as well as 2-pentylfuran from 2-nonenal. The intramolecular cyclisation of both examples is catalysed by amino acids starting from the respective 4-hydroxy-2-alkenal intermediate [26]. The results of the present study also showed that this mechanism, with an intramolecular cyclisation under the elimination of water, can be considered very likely at high temperatures, even without nucleophilic amino compounds present (Figure 4 and Figure 5).

Such heterocyclic furans and their derivatives belong to the thermally-induced process contaminants in food, such as the very prominent examples of acrylamide and chloropropanols [29,52]. However, 2-methylfuran, 2-acetylfuran, and furfural are mainly formed by thermally-induced decomposition of carbohydrates and in the course of the Maillard reaction [52,53]. In contrast, 2-alkyl furans and similar chemical structures with a longer alkyl chain have been already described in oils [54,55] and lipid-rich foods [56,57]. Alkyl furans are aroma-active compounds and can affect the flavour of food. The sensory attribute of 2-pentylfuran is described as green [58] and liquorice and beany [59], whereas 2-ethylfuran has been described as roasted [60] and sweet and coffee-like [52]. In the present study, besides 2-pentylfuran, the aldehyde heptanal was also detected during the heat treatment of 2-nonenal (Figure 4A). It is well-known as secondary lipid oxidation product and has been detected in various food such as cheese [61], meat products [62], soybean oil [63] and was therefore used as a marker for lipid oxidation [61,64]. Nevertheless, the origin of heptanal has not yet been comprehensively identified. Frankel (2012) suggested that heptanal is a degradation product of oleic acid, but he emphasized that heptanal cannot result from the typical β-cleavage [65]. Selke et al. (1977) were able to detect heptanal from triolein model systems heated up to 192 °C [66], whereas Jeong et al. (2010) could not detect heptanal during the heat treatment of oleic acid at 93 °C [67]. Other authors have identified heptanal in linoleic acid model systems [68,69]. In addition, an increased formation of heptanal in model systems and milk was demonstrated in the presence of a photosensitizer, typically for inducing the start of lipid peroxidation [68,70]. The present study showed that in addition to 2-pentylfuran, 2-nonenal can also be a precursor for heptanal. It should be mentioned that 2-nonenal is a known degradation product of linoleic acid and thus, heptanal is a tertiary degradation product of linoleic acid. The degradation of 2-nonenal by conventional lipid autooxidation and β-cleavage should led to octanal [65] instead of heptanal. Therefore, degradation by photooxidation [68] or via an epoxy intermediate [71,72] or a thermal breakage of the double bond [50] seems to be more likely. 

In summary both observed reactions, the formation of alkyl furan and the formation of aldehydes are responsible for the decrease in the formation rate of 2-alkenals demonstrated in heated rapeseed oils (Figure 2).

#### 3.3.2. Degradation of Aldehydes–Aldol Condensation Reaction

Due to the stagnation in the area of the aldehydes, especially hexanal from 30 min onwards, degradation reactions to tertiary products are be expected. In order to investigate further degradation reactions of saturated aldehydes we used hexanal and propanal as most common aldehydes from lipid oxidation and especially from linoleic and linolenic acid in model systems at 160 °C in inert paraffin oil. 

**Figure 6:**
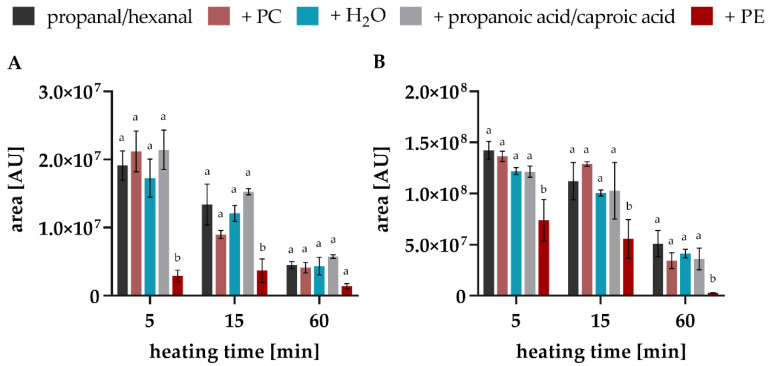
Degradation of (**A**) propanal and (**B**) hexanal in model studies with addition of PC, H_2_O, propanoic acid/caproic acid, or PE at 160 °C 5, 15 and 60 min. Statistically significant values (*p* < 0.01) within the columns are designated by different letters and refer in each case to the initial value of propanal/hexanal without the addition of further compounds (two-way ANOVA; Dunnett’s test).

To study the influence of further compounds which can be part of food matrices, model systems with additions of water, propanoic acid/caproic acid (the oxidised acid of the respective aldehydes) or PE and PC (as lecithin components) were carried out in addition to the pure aldehydes. At the beginning of the heating period, a decomposition of hexanal can be observed, especially when PE is present (approx. 50% less hexanal, Figure 6A). This effect is even stronger in the experiments with propanal (approx. 85% less propanal, Figure 6B). The complete kinetics over the time course of 60 min are shown in Figure S3 (provided in Supplementary Materials).

**Figure 7:**
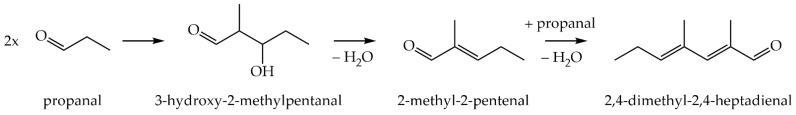
Aldol condensation reaction of propanal to the aldol adduct 3-hydroxy-2-methylpentanal; dehydration to 2-methyl-2-pentenal (dimer) and subsequent reaction to 2,4-dimethyl-2,4-heptadienal (trimer).

In addition to the degradation of aldehydes, the formation of aldol reaction products was also observed. In the experiments with hexanal, the self-aldol condensation product 2-butyl-2-octenal could be observed. Whereas in the experiment with propanal in addition to the aldol condensation product 2-methyl-2-pentenal, the addition product 3-hydroxy-2-methylpentanal and an aldol condensation product consisting of three units propanal 2,4-dimethyl-2,4-heptadienal were detected (Figure 7). 

**Figure 8:**
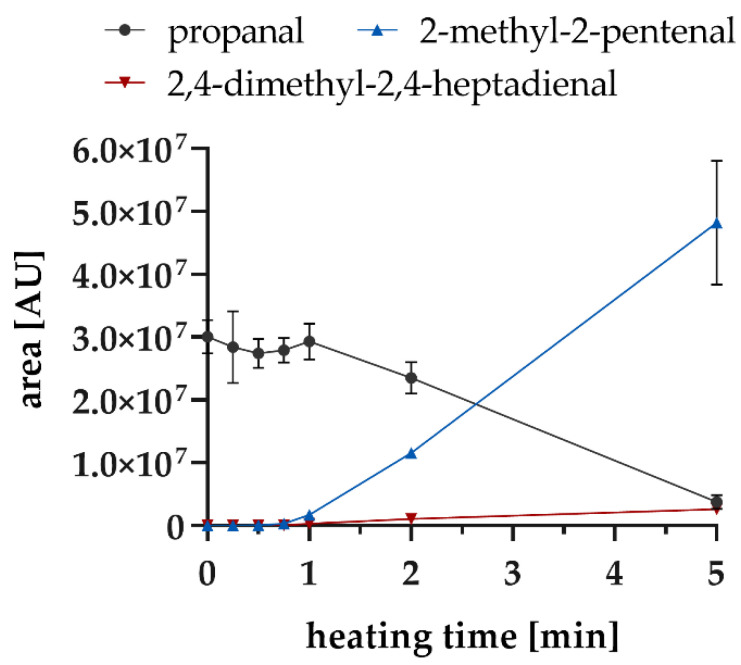
Degradation of propanal with addition of PE and the formation of the dimer and trimer aldol condensation products at 160 °C within 5 min.

The strong degradation rate of propanal and the formation rate of the aldol reaction products in the experiments with addition of PE were investigated in more detail for the first 5 min, as the degradation was greatest during this period. Figure 8 shows that from 60 s, the degradation of propanal correlates with the formation of 2-methyl-2-pentenal.

Aldol condensation products are known to act as aroma-active compounds in different foods. 2-Butyl-2-octenal has been described in e.g., orange oil [73], rice [74] pine nuts [75], and walnut oil [76]. Its sensory attributes are described as meaty [77], savoury [78], or grassy and fruity [79]. The self-aldol condensation of hexanal is usually catalysed enzymatically or under mild acidic conditions [73]. The present results showed that a thermally induced aldol condensation also takes place. Consequently, these compounds might also contribute to changes in the aroma of rapeseed oil as a result of an enhanced lipid oxidation. The aldol reaction products can be seen as tertiary lipid oxidation products because they emerge in subsequent reactions from secondary lipid oxidation products in form of various aldehydes.

## 4. Conclusions

The volatile secondary and tertiary degradation products formed during lipid oxidation are important, as they are responsible for certain (off)-flavour-attributes in food, especially at elevated temperatures. Consequently, their determination enables a monitoring of fats and oils with the possibility of identifying excessive heating. Once such methods are fully established and validated, they can be used in routine analysis. Although these methods are more laborious than rapid detection methods, they are more accepted by official food safety authorities. Obviously, they can be regarded as supplemental to the portfolio of methods for evaluating fat/oil quality, which are often focus on more simple markers or measuring polymeric lipid oxidation fractions.

However, these marker substances must be very carefully selected, as secondary volatile compounds are formed during lipid oxidation and at the same time they are also subjected to subsequent reactions. These further reactions lead to tertiary products such as alkyl furans, aldol condensation products or methyl ketones [11]. In addition, interactions with other food compounds, such as water or nucleophilic nitrogen components (e.g., phospholipids) are affecting the composition of the VOC and can enhance the formation of tertiary degradation products. However, the initial status in oils, fats, or foods must be considered, because “the age” of an oil influences the formation of tertiary products significantly as well.

Therefore, peroxides and primary or dominant secondary lipid-derived VOC are only of limited suitability as marker substances for the status of lipid oxidation. Aldehydes such as propanal and hexanal and 2-alkenals are suitable as markers for the early phase of lipid oxidation, but at an advanced stage, they are subject to aldol condensation reactions or can be oxidized further by reactive oxygen species or by thermally-induced degradation processes to 2-alkyl furans. Consequently, the detection of these tertiary degradation products (aldol condensation products, 2-alkyl furans and methyl ketones) as marker substances, is well suited to indicate advanced lipid oxidation. In general, multiple VOC, ideally from different stages of the lipid oxidation, should be taken into consideration in order to make a statement about the course of lipid oxidation and therefore about the oxidative status of oils and fat containing foods. Future studies should investigate processes at lower temperatures and storage conditions to determine the temperature influence on the formation of tertiary products from lipid oxidation and to identify unambiguous markers.

## Data Availability

The data presented in this study are available on request from the corresponding author.

## Supplementary Materials

The following are available online at https://www.mdpi.com/article/10.3390/foods10102417/s1, Figure S1: Static headspace GC-MS TIC chromatograms of ‘RO 4‘ after heating at 160 °C for 5 min (A) and 120 min (B) and ‘RO 20’ after heating at 160 °C for 5 min (C) and 120 min (D); detected peaks above detection limit are marked with numbers; Figure S2: Kinetic of selected compounds during 120 min at 160 °C; A: ‘RO 4’; B: ‘RO 20’; Figure S3: Kinetics of hexanal (A) and propanal (B) degradation with different compounds; Formation of aldol condensation products from hexanal (black lines) or propanal (red lines) and PE (C) and PC (D); Table S1: Listing of the volatile compounds formed during heating of ‘RO 4’ and ‘RO 20’ after 5 min at 160 °C; Table S2: Listing of the volatile compounds formed during heating of ‘RO 4’ after 120 min at 160 °C; u. d. under detection limit; Table S3: Listing of the volatile compounds formed during heating of ‘RO 20’ after 120 min at 160 °C; u. d. under detection limit.

## Sample Availability

Samples of the compounds are available from the authors.
