# Supplementary material for: Formation of Secondary and Tertiary Volatile Compounds Resulting from the Lipid Oxidation of Rapeseed Oil
Source: Foods. 2021 Oct 12;10(10):2417. doi: 10.3390/foods10102417 (PMC8535505; doi:10.3390/foods10102417)
Supplement: Supplementary file 1 [file foods-10-02417-s001.zip › foods-1380986-supplementary.pdf]

Table S1. Listing of the volatile compounds formed during heating of 'RO 4' and 'RO 20' after 5 min at 160 °C; u. d. under detection limit.

RO 4	retention time [min]	area [AU]	compound
1	15.218	433092	alkane
2	16.180	1825140	hexanal
3	19.752	467423	alkane
4	20.771	482210	2-heptenal
5	22.200	234825	2,4-heptadienal
6	22.308	636344	3-butenylisothiocyanate
7	22.750	277247	2,4-heptadienal
8	25.231	222149	nonanal

RO 20	retention time [min]	area [AU]	compound
1	7.304	20314931	u. d.
2	9.402	1933964	acetic acid
3	10.162	11916064	butanal
4	12.462	3692520	2-butenal
5	12.991	600654	1-penten-3-ol
6	13.590	1242816	pentanal
7	15.577	1585614	2-pentenal
8	16.536	12706509	hexanal
9	17.180	673997	u. d.
10	18.919	1253995	u. d.
11	19.323	1038388	heptanal
12	20.108	1683075	u. d.
13	20.164	707317	u. d.
14	20.556	411531	alkane
15	21.205	2859596	2-heptenal
16	21.757	120867	2-pentylfuran
17	21.918	221044	u. d.
18	22.302	243586	u. d.
19	22.672	1679288	2,4-heptadienal
20	23.226	1377288	2,4-heptadienal
21	25.759	1436667	nonanal

Table S2: Listing of the volatile compounds formed during heating of 'RO 4' after 120 min at 160 °C; u. d. under detection limit.

number	retention time [min]	area [AU]	compound
6.310	5.258	208045823	u. d.
6.414	6.586	471223717	u. d.
6.827	6.741	628207847	u. d.
9.483	6.951	79242812	acetic acid
9.999	7.113	32118663	u. d.
12.190	7.236	358562186	u. d.
12.599	9.592	74046129	1-penten-3-ol
13.044	10.042	32021580	2-ethylfuran
13.227	10.199	100586308	pentanal
15.158	10.331	361826439	alkane
16.166	12.543	232381676	hexanal
17.475	12.999	3713132	3-hexen-2-one
17.618	13.402	2206045	pentanoic acid
17.856	13.579	22954149	2-hexenal
18.562	14.076	8284717	2-heptanone
18.903	15.247	24867135	heptanal
19.357	15.519	11085452	u. d.
19.735	15.841	4456628	alkane
20.364	16.199	7785309	alkane
20.748	16.533	90820327	2-heptenal
21.285	16.913	20352050	2-pentylfuran
21.402	17.022	4403603	2-octanone
21.819	17.172	20145136	octanal
22.174	17.867	11213459	2,4-heptadienal
22.717	17.987	48575809	2,4-heptadienal
23.939	18.253	12465029	2-octenal
25.200	18.532	53935826	nonanal
27.727	18.704	3896741	2-nonenal
32.196	18.969	13399598	2-decenal
34.136	19.214	2055702	2,4-decadienal
35.457	19.317	8606954	2,4-decadienal
37.245	19.582	6285718	2-undecenal

Table S3: Listing of the volatile compounds formed during heating of 'RO 20' after 120 min at 160 °C; u. d. under detection limit.

number	retention time [min]	area [AU]	compound
1	5.258	45805732	u. d.
2	6.586	132137245	u. d.
3	6.741	294294334	u. d.
4	6.951	139231867	u. d.
5	7.113	200586875	u. d.
6	7.236	304887095	u. d.
7	9.592	47019360	acetic acid
8	10.042	11199853	u. d.
9	10.199	11476315	butanal
10	10.331	36453040	u. d.
11	12.543	319152022	alkane + 2-butenal
12	12.999	73688665	1-penten-3-ol
13	13.402	41776137	2-ethylfuran
14	13.579	81020043	pentanal
15	14.076	1209789	u. d.
16	15.247	2632242	u. d.
17	15.519	302285253	2-pentenal
18	15.841	1660668	alkane
19	16.199	2328220	u. d.
20	16.533	168238250	hexanal
21	16.913	140554	u. d.
22	17.022	2345842	u. d.
23	17.172	1645792	u. d.
24	17.867	3652260	3-hexen-2-one
25	17.987	2140053	pentanoic acid
26	18.253	18150801	2-hexenal
27	18.532	324192	u. d.
28	18.704	280813	u. d.
29	18.969	7737926	2-heptanone
30	19.214	3106538	u. d.
31	19.317	20464090	heptanal
32	19.582	1010690	u. d.
33	19.785	9411576	alkane
34	19.850	2341203	alkane
35	20.160	4264655	u. d.
36	20.559	1456323	alkane
37	20.741	6784252	hexanoic acid
38	20.990	1787496	alkane
39	21.198	60641529	2-heptenal
40	21.600	1945961	1-hepten-3-one
41	21.750	16551114	2-pentylfuran
42	21.871	5870861	2-octanone
43	22.294	13065577	octanal
44	22.661	13815513	2,4-heptadienal
45	23.217	47411653	2,4-heptadienal
46	23.723	575678	3-octen-2-one
47	23.985	339672	u. d.
48	24.129	885834	alkane
49	24.470	9602664	2-octenal
50	25.417	701011	4-hydroxy-2-hexenoic acid lactone
51	25.559	622424	u. d.
52	25.755	38218384	nonanal
53	26.178	1787143	u. d.
54	27.409	495536	u. d.
55	27.742	395144	u. d.
56	28.344	2888643	2-nonenal
57	29.583	439345	u. d.
58	29.857	279811	decanal
59	32.183	286036	u. d.
60	32.893	9902856	2-decenal
61	33.399	407580	u. d.
62	34.869	1660861	2,4-decadienal
63	36.208	7169564	2,4-decadienal
64	38.016	5087844	2-undecenal
65	39.901	95486	2,4-undecadienal

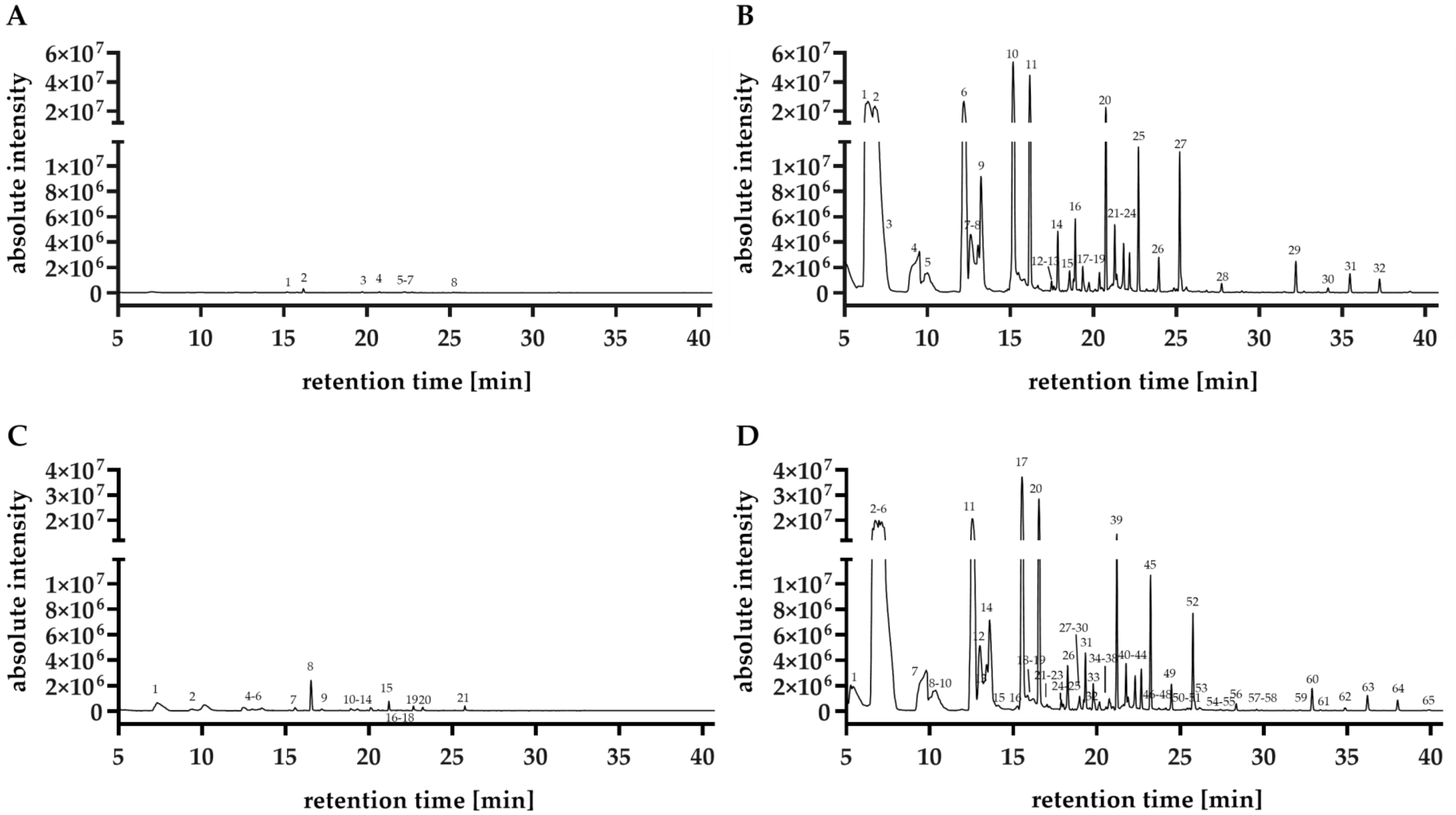

Figure S1. Static headspace GC-MS TIC chromatograms of 'RO 4' after heating at 160 °C for 5 min (A) and 120 min (B) and 'RO 20' after heating at 160 °C for 5 min (C) and 120 min (D); detected peaks above detection limit are marked with numbers.

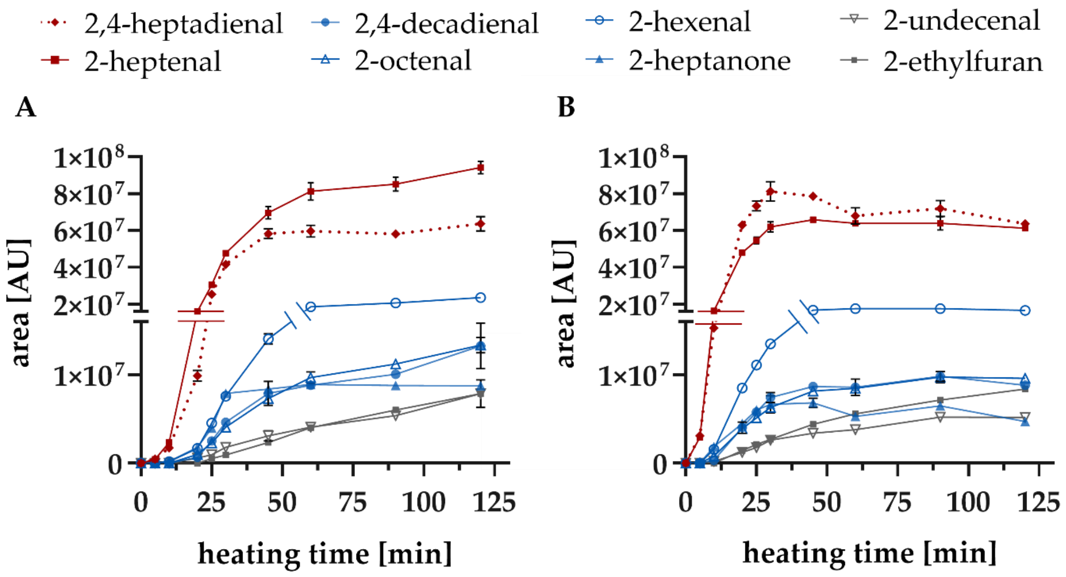

Figure S2. Kinetic of selected compounds during 120 min at 160 °C; A: 'RO 4'; B: 'RO 20'.

Based on their formation and degradation rates individual VOC from lipid oxidation can be classified into early phase (figure S1 red lines), advanced phase (blue lines) and late phase degradation products (grey lines). VOCs of early phase are 2,4-heptadienal (derived from 18:3 fatty acid) and 2-heptenal (18:2). 2-hexenal (18:3); 2,4-decadienal (18:2) 2-heptanone and 2-octenal (18:2) can be classified into the advanced phase and 2-undecenal (18:1) and 2-ethylfuran into late phase degradation products. For a better overview, the kinetics of eight selected compounds are shown in figure S1.

Like mentioned before, the oxidation rate of a fatty acid increases with increasing number of double bonds. Interestingly, degradation products from the triple unsaturated linolenic acid (18:3) such as 2,4-heptadienal and products from the double unsaturated linoleic acid (18:2) such as 2-heptenal can be detected in the early phase. The same can be observed in advanced phase. 2-hexenal derived from linolenic acid, whereas 2,4-decadienal and 2-octenal derived from linoleic acid. Thus, at 160 ° C linolenic acid is not attacked preferentially at the beginning, as is the case at lower temperatures [3]. Degradation products of oleic acid such as 2-undecenal and tertiary 2-alkyl furans could only be detected in the late phase.

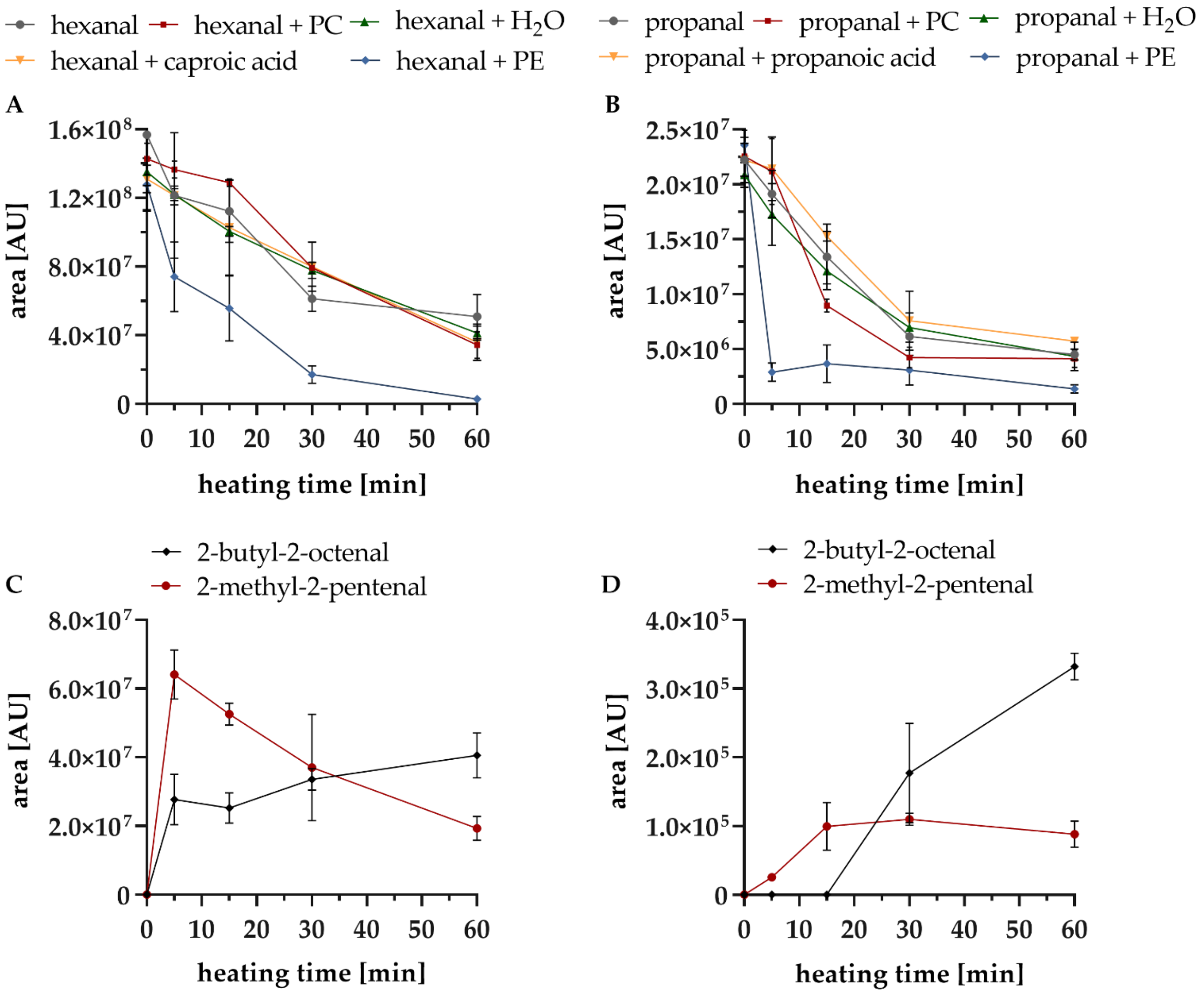

Figure S3. Kinetics of hexanal (A) and propanal (B) degradation with different compounds; Formation of aldol condensation products from hexanal (black lines) or propanal (red lines) and PE (C) and PC (D).
